# Vascular Effects of Photodynamic Therapy with Curcumin in a Chorioallantoic Membrane Model

**DOI:** 10.3390/ijms20051084

**Published:** 2019-03-02

**Authors:** Hilde Harb Buzzá, Lucas Cruz Fialho de Freitas, Lilian Tan Moriyama, Ramon Gabriel Teixeira Rosa, Vanderlei Salvador Bagnato, Cristina Kurachi

**Affiliations:** São Carlos Institute of Physics, University of São Paulo (USP), P.O. Box 369, 13560-970 São Carlos, São Paulo, Brazil; lucas.cff05@gmail.com (L.C.F.d.F.); lili@ifsc.usp.br (L.T.M.); ramongabriel.tr@gmail.com (R.G.T.R.); vander@ifsc.usp.br (V.S.B.); cristina@ifsc.usp.br (C.K.)

**Keywords:** photodynamic therapy, curcumin, vascular effect, PDT, photosensitizer, chorioallantoic membrane

## Abstract

Photodynamic Therapy (PDT) is a treatment that requires light, a photosensitizing agent, and molecular oxygen. The photosensitizer is activated by light and it interacts with the oxygen that is present in the cellular microenvironment. The molecular oxygen is transformed into singlet oxygen, which is highly reactive and responsible for the cell death. Therefore, PS is an important element for the therapy happens, including its concentration. Curcumin is a natural photosensitizer and it has demonstrated its anti-inflammatory and anti-oxidant effects that inhibit several signal transduction pathways. PDT vascular effects of curcumin at concentrations varying from 0.1 to 10 mM/cm^2^ and topical administration were investigated in a chick Chorioallantoic Membrane (CAM) model. The irradiation was performed at 450 nm, irradiance of 50 mW/cm^2^ during 10 min, delivering a total fluence of 30 J/cm^2^. The vascular effect was followed after the application of curcumin, with images being obtained each 30 min in the first 3 h, 12 h, and 24 h. Those images were qualitatively and quantitatively analyzed with a MatLAB^®^. Curcumin was expected to exhibit a vascular effect due to its angio-inhibitory effect. Using curcumin as photosensitizer, PDT induced a higher and faster vascular effect when compared to the use of this compound alone.

## 1. Introduction

Curcumin is a compound derived from the turmeric root that was traditionally used for coloring and flavoring food. Several in vitro and in vivo studies have demonstrated its anti-inflammatory [1], anti-oxidant, ant-infectious [2], and anti-carcinogenic effects, besides the chemopreventive activity [3] and positive results in the treatment of obesity and diabetes [4]. However, its clinical application is restricted due to the low solubility in water and bio-availability [5], despite several efforts to improve it [6]. Curcumin shows no toxic effects in human, including high doses, with 6 g/day during seven weeks [7] or 12 g/day [6] and efforts have been made as topical administration and encapsulation in nanoparticles [8,9].

It has also been potentially tested in the treatment of cancer of pancreas and colon, psoriasis, and Alzheimer disease, as it reaches molecules, such as growth factor, transcription factors, and cytokines that are involved in the etiology of several diseases [4]. One of the most important actions of curcumin is the inhibition of induction pathways of pro angiogenic factor—FGF-1—property that can be used in therapies that aim towards the inhibition of new blood vessel formation [10].

The scientific community has been engaged in the development of new therapeutic modalities and in the improvement of the existing ones for several diseases, including vascular disorders and cancer treatment [11]. Photodynamic therapy (PDT) is one option with a localized effect that, in some cases, can be an alternative to surgical procedures and it has high potential to minimize their side effects [12]. Light at specific wavelength interacts with a photosensitive compound (photosensitizer, PS), previously administered, inducing a series of photochemical reactions, mainly with molecular oxygen in the cellular microenvironment, which yields reactive oxygen species and may lead tissue/cell to death [13,14]. 

Curcumin has also shown to be an efficient PS, [15,16] and it has been used in tests with several applications of PDT, especially the inactivation of microorganisms [17,18,19,20,21]. Due its absorption peak in the blue region, curcumin may constitute a good PS option for superficial diseases [16,17,18,22]. The curcumin that was used in this study has been tested in pre-clinical and clinical trials for application in several diseases, showing its efficacy as a photosensitizer [23,24,25,26].

PDT may also cause the shutdown of blood vessels, since it can be localized within or at the membrane of the endothelial cells, and it has already been used to treat vascular diseases [27]. Among the vascular deformities, there are port wine stains (PWS) and hemangiomas, which affect more than 10% of all children worldwide in the head and neck areas. PWS are superficial capillary dilatations of the skin, at depths from 100 to 1000 μm. Hemangiomas are tumors that arise from vascular endothelial cell proliferation, and most hemangiomas are benign. However, they can cause some serious health problems, such as atrophy, pigmentation defects, and, due to cartilage damage, resulting in deformations of the ear or nose, in addition to possible psychological damage. Common treatment methods for such deformations include the application of corticosteroids, pulsed dye laser therapy, and surgical excision. More recently, Photodynamic Therapy has been tested for the treatment of these lesions [16,28].

The Chorioallantoic Membrane (CAM) membrane model in chicken eggs is formed by the fusion of the allantois and chorion and it has the function of a respiratory system in avian embryos [29]. It is a transparent membrane through which blood vessels and structural changes can be visualized. With this model, it is possible to conduct studies with many parameters that are linked with PDT and, therefore, it is an option to study vascular effects. The CAM model can be used to understand how a compound acts on the vascular endothelium, since it enables direct access to blood vessels, besides being a cheap, simple, and easy model for laboratorial implementation [30,31]. It has been considered as an alternative method for the use of animal models, since there are no neural receptors until the 14th day of embryo development, which results in no pain being induced to the embryo [32].

Therefore, the present manuscript addressed the joint effect of curcumin as an anti-angiogenic compound and as a photosensitizer for PDT in the CAM model.

## 2. Results

We investigated the effects of curcumin in the CAM model. As hundreds of images were acquired, not all data has been shown, but only the most representative ones. A similar behavior among the CAMs was observed within the same group. Besides this, both types of curcumin that were tested displayed the same behavior, when compared the same condition. For this, all analyses considered the membranes of both curcumin in the same group. 

When only light was applied to the CAM, testing all the irradiances and light doses (groups L1–L5), no major macroscopic changes were observed in the vascular network, which suggest that light under the evaluated parameters was not able to induce any vascular damage.

**Figure 1:**
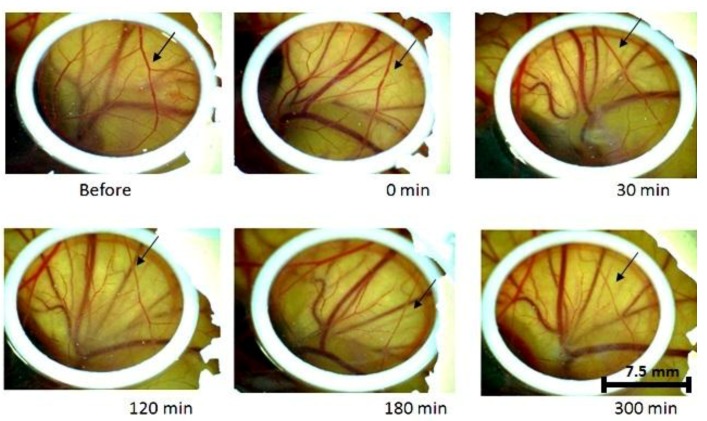
Only Curcumin (0.1 mM) effect over the time without light. The arrows point out a vessel that became thinner over time.

**Figure 2:**
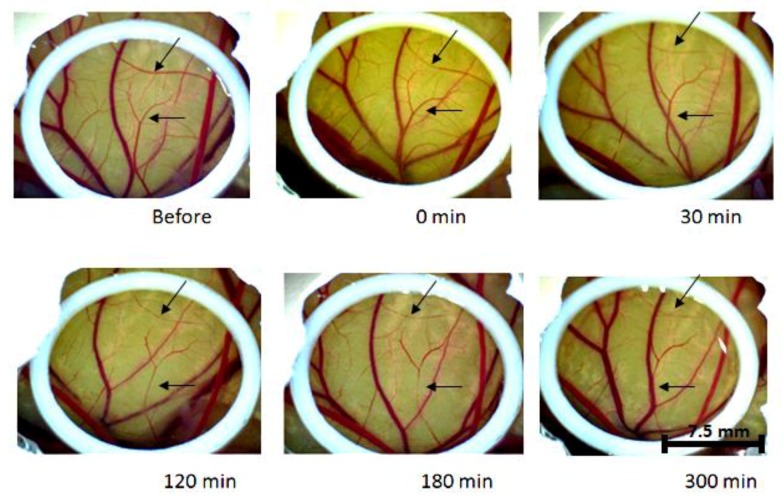
Only Curcumin (10 mM) effect over the time without light. The arrows point out vessels that became thinner over time.

When only curcumin was applied to the CAM, for all concentrations tested (0.1, 0.33, 0.5, 1, and 10 mM/cm^2^), some changes were observed, resulting in a decrease in the blood vessels diameter, confirming the anti-angiogenic effect of the curcumin itself. This vascular response that was induced by curcumin was more evident with the increase of concentration, as shown in Figure 1 and Figure 2, when comparing 0.1 mM/cm^2^ and 10 mM/cm^2^ (groups C1 and C6, respectively). In Figure 1, the arrows point out a vessel that became thinner after curcumin application. After 180 min, there is a more prominent shrinkage.

When increasing the concentration up to 10 mM/cm^2^, the effect of the curcumin in the vessels was faster, as shown in Figure 2. Thirty minutes after the curcumin incubation, the shrinkage of the vessels started to be evident, and 300 min after the incubation, some of the vessels had collapsed. This was an expected result, since a higher concentration of curcumin means a higher availability of the compound to induce any effect. However, the higher concentration does not enable us to keep the eggs 24 h after application, because the embryos died and, to observe the long period effect after curcumin, a concentration up to 1 mM was tested.

**Figure 3:**
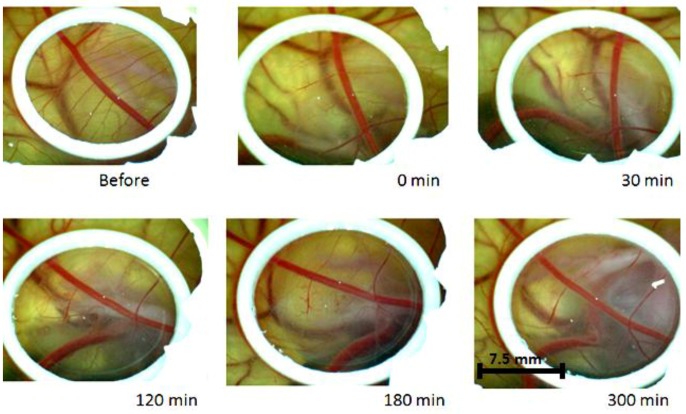
Photodynamic therapy (PDT) response with curcumin concentration that 0.1 mM over the time with total dose of 30 J/cm^2^.

Using curcumin as a photosensitizer for PDT, the obtained results indicate that the vascular effect is potentialized on the vessels. Figure 3 shows the destruction of the vessels by PDT using the curcumin concentration of 0.1 mM (group PDT1). A faster effect of the vessels shrinkage was immediately observed after the illumination (0 min), when the smaller vessels started to collapse.

The response to the PDT was more evident when a higher concentration of curcumin was used. Immediately after PDT with curcumin 10 mM, the shrinkage of the smaller vessels was already observed and 30 min after, almost the entire vascular network, except the main vessel, had collapsed. With the images, it was possible to estimate the diameter of vessels, based on the ring dimensions, and this analysis showed effect in vessels higher than 70 μm. Vessels of big diameter (about 400 μm) presented a reduction of up to 30%, after PDT.

**Figure 4:**
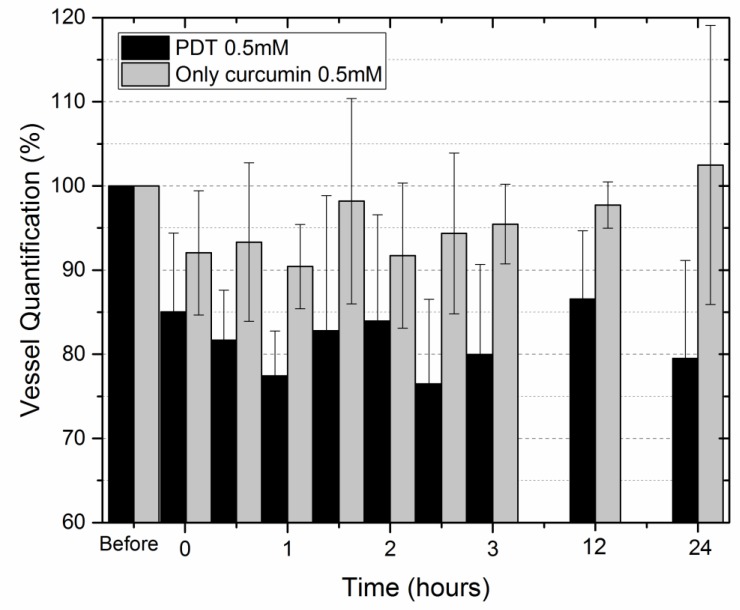
Quantification of blood vessels normalized with the image without curcumin comparing the effect of only curcumin and PDT for concentration of 0.1 mM and 30 J/cm^2^.

An image processing for the quantification of these effects was performed to compare the effect for different concentrations of curcumin and the comparison between the same concentration with and without irradiation. The graph of Figure 4 shows the area percentage of vessels normalized by the value in the first image (before the PS application) and presents the difference between the groups with only Curcumin and PDT with 0.5 mM (groups C3 and PDT3, respectively). A reduction of about 20% was observed after 24 h, in contrast with only curcumin, since there was the revascularization.

**Figure 5:**
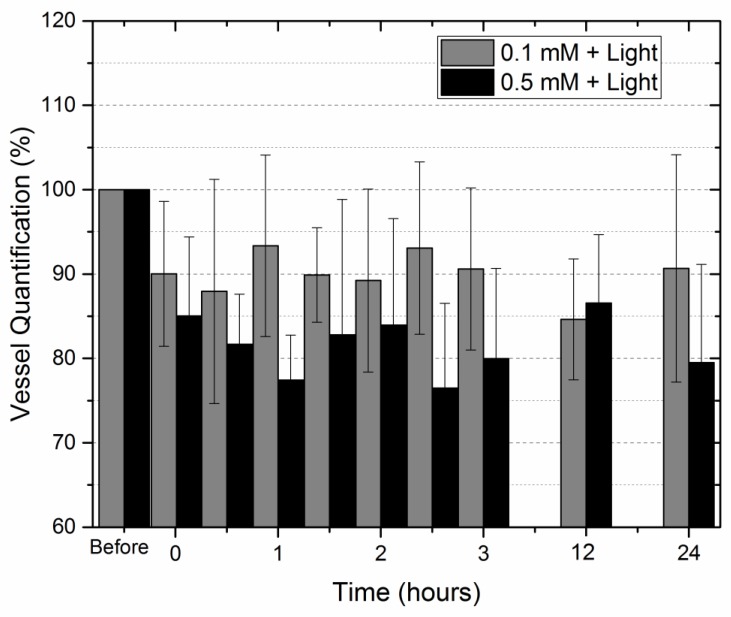
Quantification of blood vessels normalized with the image without curcumin comparing the effect of PDT with concentration of 0.1 mM and 0.5 mM and both with 30 J/cm^2^.

The increase in curcumin concentration resulted in an increased effect on the PDT groups. The graph of Figure 5 shows this behavior, when comparing the PDT groups with 0.1 mM (group PDT1) and 0.5 mM (group PDT3) up to 24 h after illumination.

With all the values obtained, a statistical analysis was performed and the level of significance was always satisfactory (*p* > 0.05).

## 3. Discussion

Although the literature reports the angiogenic effects of phototherapies, which only means the use of light [33,34,35], even in CAM, with the increase of blood vessels number [34], we did not observe the changes caused by light in our experiments, probably by the chosen parameters. The angiogenesis that was promoted by phototherapies was evident at several days after illumination and, in our experiments, the vascular response was monitored for only 24 h. 

A small effect was observed in almost all groups, because it was necessary to choose parameters that enable the survival of the embryo and, if the damage was severe, the vascular network could be destroyed and, consequently, the embryo would die. 

In the groups C1, C2, C3, C4, C5, and C6, blood vessels shrinkage was observed after 40 min of contact of curcumin with CAM. Even for the lowest concentration used, the small vessels of the CAM presented a visible decrease in diameter. The shrinkage effect was faster for higher concentrations, and this response can be associated with the fact that the viability of the compound was higher, which induces a faster response of the endothelial cells. The acquired images showed that, 5 h after the incubation with curcumin, the general aspect of the CAM was similar in all of the groups, i.e., most part of the small vessels collapsed and the thicker vessels showed slightly shrinkage, but did not collapse. After 24 h, the curcumin groups have presented the recovery of most vessels.

PDT with other photosensitizers, such as hematoporphyrin derivate (Photogem^®^) and cholorins (Photodithazine^®^), as tested in the CAM model, proved to be extremely efficient in the reduction of vascular network and it reached over 60% of reduction in the number and size of vessels, using similar total doses (30 J/cm^2^) [36]. As shown in the results presented here, other results in the literature confirmed that light amplifies the effect of curcumin [37,38]. Immediately after the irradiation, thinner vessels collapsed in all of the experimental PDT groups. 

The shrinkage of thicker vessels mainly occurred in the PDT6 group, where 10 mM/cm^2^ of curcumin solution was applied and 30 J/cm^2^ was delivered. For higher concentrations, however, following the effect after 24 h was impossible due to severe damage and the consequent death of the embryo. Studies with dorsal window chamber model in mice allow to use higher doses of light (from 100 to 500 J/cm^2^) and total shutdown of vessels, since it is possible to keep the analysis of experiment, without compromising animal life [39].

Due the absorption peak of curcumin is at the blue region (440 nm), which has low penetration in biological tissue, it has been used only in superficial clinical applications, such as onychomycosis and oral decontamination, but, related to vascular diseases, it can be used in superficial vascular diseases, such as port-wine stain [28].

With the increase of curcumin concentration from 0.1 to 10 mM, the vascular damage was observed immediately after illumination, as was possible to confirm with the quantification analysis of the blood vessels, where all the concentrations kept the reduction around 30%. The literature shows that increasing the photosensitizer concentratio, also increases the effect of cell death in other tests besides CAM, such as increasing the apoptotic population for tumor cells [40] or greater antimicrobial activity [41].

In the CAM model, the response of photodynamic therapy is usually proportional to the dose of light delivered [42]. Tests using CAM are performed with doses of light that are in the range between 10 and 50 J/cm^2^, which is sufficient for the complete occlusion of blood vessels. There are some exceptions for specific photosensitizers. For example, the topical application of chlorines showed a larger occlusion at 10 J/cm^2^ doses with irradiance of 10 mW/cm^2^ when compared to 15 and 20 J/cm^2^ (with 15 and 20 mW/cm^2^, respectively). This difference for high doses may be related to the depletion of oxygen or faster photobleaching [43].

The increase in the concentration of photosensitizers also proportionally influences the photodynamic action, in the great majority of cases and, the class of photosensitizer used may change this behavior. Verteporphyrins, for example, are widely used for vascular applications and show total occlusion in the CAM model, reaching larger vessels with increased drug concentration (with diameters of about 70 μm) in small treated areas. New photosensitizers have reached vessels up to 30 μm [44]. 

Our study, therefore, has shown satisfactory results, since curcumin was used with similar doses to those that are found in the literature, with the occlusion of vessels that were bigger than 70 μm in much larger areas of treatment. The use of porphyrin derivatives, such as PpIX, using 20 J/cm^2^ did not show occlusion after 120 min of PDT. The same encapsulated photosensitizer showed an effect 60 min after PDT reaching vessels up to 70 μm [45]. If we compare with our result, while applying similar doses of light, curcumin started to present occlusion immediately after light and photosensitizer application, in vessels with similar diameter. 

When the dose increase does not correspond to an increase in the photodynamic effect, this may often be related to the state of aggregation of the molecule, which can occur when there are high concentrations of the photosensitizer [46].

The analysis of the most common vascular effect that was reported in the literature is the application of a score ranging from 0 to 5, and this value increases as the affected vessels are of bigger diameter. On this scale, zero means the occlusion and 5 means total closure. However, these methods are used for the analysis of small areas—approximately 0.19 cm^2^ [45] or even less than 2 mm^2^ [46]—corresponding to few blood vessels. When the analysis presents an area of 1.76 cm^2^, as in the case of the present study, with a larger number of vessels, this adopted method may not show specific results. Therefore, image processing using computational tools, with the calculation of the area that is occupied by the blood vessels here demonstrated, presents a new possibility and is more comprehensive for the study of the vascular effect from different therapies that are related to blood vessels.

Our findings show the vascular response to the application of only curcumin and to PDT as a function of time. When the CAM was monitored for 5 h, the vascular damage that was induced by PDT was much more apparent than when only curcumin was used. After 24 h, a reduction of vessels was still present, mainly in PDT groups as compared to only curcumin.

The literature using curcumin as PS for inducing vascular occlusion in the CAM model, or even to analyze vascular effect, is very poor. In the chorioallantoic membrane, Duse et al. showed the effect of liposomal formulations with Curcumin as photosensitizer; however, there is not a comparison with the anti-angiogenic effect of this compound [47].

With the objective of increasing the discussion about vascular effect, the present study has demonstrated that topical application of curcumin solution can potentially photosensitize blood vessels in the CAM model. The vascular occlusion that is induced by curcumin incubated for 40 min can be enhanced by irradiation at 450 nm. The vascular occlusion degree increased with drug concentration and light dose. These results may help to understand the individual vascular effect and improve PDT planning using curcumin as a photosensitizer.

## 4. Materials and Methods

### 4.1. CAM Model

Chicken eggs were obtained from a local producer (A´DORO S.A., São Carlos/SP, Brazil). According to the procedure that was previously described [8], the eggs were cleaned with 70% alcohol on the first day of embryo development (ED1) and kept in a humid incubator at 37.7 °C, in constant slow rotation motion—half period each 30 min. On the ED3, a small hole was produced with a needle in the shell to remove from 2 to 3 mL of albumin and a 2 cm^2^ window was then opened in their thinner part and sealed with adhesive tape. The eggs remained in the incubator until the 11th day, when the vessels reached the appropriate size to analyse, which means that almost all of the vessels have a diameter above 80 μm. All of the procedures were conducted inside a laminar flow hood to avoid contamination. Institutional Animal Care and Use Committee of Sao Carlos Institute of Physics (protocol number 9/2014) and Institutional Animal Care and Use Committee of Federal University of Sao Carlos (protocol number 068/2012) approved the experimental protocol.

### 4.2. Experimental Groups

Fifty eggs were divided into 13 groups with at least three eggs per group, as described below.

Curcumin powder from two companies (PDT Pharma LTDA, Cravinhos, Brazil and Vetec Quimica, Sigma Aldrich–Merck KGaA, Darmstadt, Germany) was tested. Each one was diluted in 1% DMSO and 99% ethanol for a stock solution with 100 mM and sterile saline solution (0.9% NaCl) was added for obtaining final concentrations. A Teflon^®^ ring of 1.5 cm diameter (and therefore, 1.76 cm^2^) was used to limit the curcumin solution on the CAM and the area of irradiation and analysis. Two hundred microliters of curcumin solution in each concentration were placed inside the ring, resulting in a concentration that is distributed in a specific area and shown as 0.1, 0.33, 0.5, 1.0, 5.0, and 10 mM/cm^2^. After 40 min of incubation, the solution excess was removed while using a micropipette and the experiments were conducted.

A homemade blue LED device emitting at 450 nm was used as the irradiation source and it was placed in a support to fix the distance between the CAM and the emission tip, such that the irradiation spot would uniformly match the Teflon^®^ ring diameter.

To only evaluate the effect of light, no curcumin solution was applied (groups L1, L2, L3, L4, and L5) to the control group. Similarly, to only evaluate the effect of curcumin solution in different concentrations, no irradiation was performed (groups C1, C2, C3, C4, C5, and C6).

After some tests to define the best parameters of irradiation, the irradiance was varied as 10, 12, 50, and 60 mW/cm^2^ during 5 and 10 min. For the PDT groups, the parameters were set as 50 mW/cm^2^ of irradiance and irradiation time was of 5 or 10 min, which resulted in 30 J/cm^2^ and 15 J/cm^2^ light fluency, respectively (groups PDT1, PDT2, PDT3, PDT4, PDT5, PDT6, PDT7, PDT8, and PDT9).

A summary of the experimental groups is provided in Table 1.

### 4.3. Evaluation of Vascular Response—Analysis of Images

The PDT outcome on CAM was evaluated through images. A USB Digital Microscope^®^ (AVANTGARDE, China) was used and all of the images were captured every 30 min after PDT irradiation until 3 h. To verify whether the vessel damage was long-term, images were performed after 12 h and 24 h of PDT irradiation. Firstly, a qualitative analysis was performed for the observation of the vascular network over the time and the behavior of the number or diameter of blood vessels was observed. 

With the use of ImageJ^®^ software (NIH, Bethesda, Maryland, USA), the diameter of vessels was measured, using the ring diameter as reference.

After that, a semi-quantitative analysis was performed using a MATLAB^®^ (The MathWorks, Natick, Massachusetts, USA) algorithm. Since the blood strongly absorbs in the 500–600 nm spectral region, the green channel of the images were used to allow an automatic detection of the vessels. To do this, a convolution was performed on the eight-bit green channel matrix with a disk mask with 30 pixels of radius. This process basically generates an image where the value of each pixel is the average of all the pixels inside a disk within the given radius. As the blood vessels have lower green values in the images, the convoluted image will tend to have higher values on the green channel than the original one in the region of vessels and lower than the original in the region outside the vessels. The radius could be adjusted according to each image to guarantee the best image reading.

A binary matrix was determined and it could be produced with a high correlation with the vessels. This matrix was calculated by evaluating the boolean test that is described by the following inequation:

Convoluted Image > α*Original Image(1)

where the best value of α was empirically determined to be 1.05. This procedure produces a binary image with high noise ratio, which was partially filtered off from the produced binary image by removing regions containing less than 1000 connected to four neighbors.

**Figure 6:**
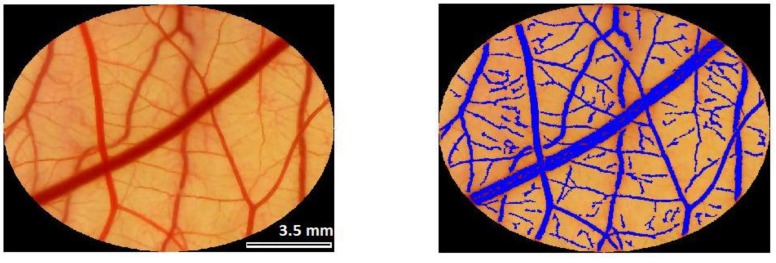
Processing the Chorioallantoic Membrane (CAM) image with MATLAB^®^ routine.

Finally, a region of interest (ROI) was manually selected for each image and the ratio between the area of the vessels (calculated from the binary image) inside this ROI was calculated. Figure 6 shows the ROI in an image that was obtained with the camera and the overlapping image resulted of the routine. This value was tracked for each image in the time sequence and was used to evaluate the treatment response. A percentage of this value was calculated, always comparing with the control image (before any procedure) and the average among these values of all eggs per group was computed and plotted in a graph.

With the values obtained with the image processing, a statistical analysis was performed using the one-way analysis of variance.

## 5. Conclusions

Curcumin is a compound that has a good vascular effect without the use of light. However, when applied light, this effect is potentiated, with the elimination of small vessels immediately after illumination and the diameter reduction of all vessels. The increase in curcumin concentration directly results in the increase of this vascular effect, presenting faster reduction, and in a larger number of vessels. The processing of the images to count the total area of occupied vessels proved to be a great form of analysis, especially in the treatment of big areas with large numbers of blood vessels. Thus, with a quantitative analysis, the potential use of curcumin for superficial vascular diseases became even more evident.

## Figures and Tables

**Table 1:** Description of the experimental groups.

Group	[Curcumin] (mM/cm^2^)	Light Irradiance (mW/cm^2^)	Light Fluence (J/cm^2^)
L1	-	10	6
L2	-	12	7.2
L3	-	50	15
L4	-	50	30
L5	-	60	36
C1	0.1	-	-
C2	0.33		
C3	0.5		
C4	1		
C5	5		
C6	10		
PDT1	0.1	50	30
PDT2	0.33		
PDT3	0.5		
PDT4	1		
PDT5	5		
PDT6	10		
PDT7	0.1	50	15
PDT8	0.5		
PDT9	1		
